# Supplementary material for: Acute respiratory infection rates in primary care anticipate ICU bed occupancy during COVID-19 waves
Source: PLoS One. 2022 May 4;17(5):e0267428. doi: 10.1371/journal.pone.0267428 (PMC9067638; doi:10.1371/journal.pone.0267428)
Supplement: Attachment — Submitted filename: Response to Reviewers.pdf [file pone.0267428.s001.pdf]

Ref.: PONE-D-21-02244, entitled "Acute Respiratory Infection Rates in Primary Care Anticipate ICU Bed Occupancy During COVID-19 Waves", Guillen M, et al., revised version

Reply to the Editor and the Reviewer

We thank you for your valuable comments, which have contributed to clearly improve the quality of the study. Here is a summary of how we have addressed your comments and the changes introduced.

The Editor: "*The present investigation is well designed and written. However, the main limitation is represented by the fact that data are somewhat old (2020). We suggest the Authors to clearly explain why and how their findings are still clinically useful*."

Authors' reply to the Editor:

The original data timeframe turned quite old because the peer review process was unusually prolonged exceeding 14 months. Initially, data covered from March 1, 2020 to January 20, 2021 (the initial draft was submitted on January 21, 2021). In the present revised version, we have included an additional Table showing results extended until March 27, 2022.

We include an additional Table showing that the anticipation lag (around 17 days) has remained stable until the Omicron variant appeared. We expect that in future outbreaks similar analyses will help anticipating ICU occupancy and this is the reason why we believe that our findings are clinically relevant.

Changes included in the manuscript:

Table 2 is inserted showing a monthly rolling window with an estimate of anticipation lag (in days) covering until March 2022 with their corresponding confidence intervals.

The following sentence has been included in the discussion section. "*This method has been successfully implemented by the local authorities. When Primary Care diagnoses of ARI increased (or decreased), subsequent ICU occupancy increase (or decreased) followed about three weeks later. This neat indicator did not depend on recorded infection rates (which could be contingent on test availability) and was only challenged when Primary Care facilities became fully saturated during the Omicron variant wave from December 2021 to January 2022. We expect that in future outbreaks similar analyses will help anticipating ICU occupancy.*"

Following your suggestion, we have made our data available in **protocols.io** (http://dx.doi.org/10.17504/protocols.io.36wgq7de5vk5/v1 and we have also updated the data in our own website http://www.ub.edu/rfa/R/covid)

Two more references were included to address one of the Reviewer's comments and the reference below was updated, because the paper has been recently published.

> Chavarria-Miró G, Anfruns-Estrada E, Martínez-Velázquez A,et al. Time-evolution of SARS-CoV2 in wastewater during the first pandemic wave of COVID-19 in the metropolitan area of Barcelona. Appl. Environ. Microbiol, 2021;87(7):e02750-20. doi: 10.1128/AEM.02750-20. [PMID: 33483313].

Reviewer: "*This is an important question but it has also been informally understood since the beginning of the pandemic that outpatient cases ◊ hospitalization 2 weeks later ◊ death 4 weeks later (roughly). That said, they provide specificity for this general concept. Also, by tying it to GP visit they establish a somewhat uniform definition of outpatient severity (i.e. asymptomatic or very mild cases wouldn't visit GP, generally).*

I have some concerns about immediate relevance given that the data are in pandemic terms quite old (2020). On the other hand, the concept is an important one and I would therefore strongly support publication."

Authors' reply to the Reviewer:

We appreciate very much your positive comments regarding the scientific interest of the study. As noted in our reply to the Editor, the reason why the data timeframe was outdated can be explained by the long time taken in the peer review of the manuscript over 14 months. We honestly were much worried about such a delay that was out of our control. Following your suggestion, we have now extended the data up to March 27, 2022. The conclusions are very similar to those we presented in the initial manuscript, except when the Omicron variant wave started.

Changes included in the manuscript:

We have included a new Table showing the anticipation lag dynamics until March 2022.

Reviewer: "*Introduction*
A nice, concise description of what is known and the problem. Very well written.
Methods
Good description of patient population, appropriate to only use index visits for ARI with PCR confirmed COVID-19. Appropriate to include positive rapid antigen as specificity is high for those tests.
US has 27 ICU beds/100,000, so this should be addressed in the Discussion; applying this to other health systems will require consideration of the local context. Criteria for hospital and ICU admission may also vary. And, reporting requirements vary by country. This makes sense in a proper organized national health system, but not in places like my country which lack a true health system. Unfortunately.
Adjustment for holidays and weekends and day of week effects is important. Not being a biostatistician I am not able to judge whether your approach was appropriate, but it seem to be so."

Authors' reply to the reviewer:

We agree with your comments. We have now included the number of ICU beds /100,000 in the US. We also note that applying our approach to other healthcare systems requires consideration of the local context, criteria for hospital and ICU admission and reporting protocols. Two references for the number of ICU beds per capita in the US have been cited.

> Organisation for Economic Co-operation and Development (OECD). Intensive care beds capacity. 2018 Available from: https://www.oecd.org/coronavirus/en/data-insights/intensive-care-beds-capacity [last access April 1, 2022]

Society of Critical Care Medicine. United States Resource Availability for COVID-19. 2020 Available from: https://www.sccm.org/Blog/March-2020/United-States-Resource-Availability-for-COVID-19 [last access April 1, 2022]

Changes included in the manuscript:

The following sentence has been included in the discussion section. "*We also note that applying our approach to other healthcare systems requires consideration of the local context, criteria for hospital and ICU admission and reporting protocols. For example, in the US, there are 30 ICU beds/100,000 approximately [two new references], which triples the rate in the region analyzed here."*

Reviewer: "*Results*
Again, very concise. Was the 17-day lag consistent throughout the study period? Also, this research was done with the ancestral variant. We are now on to omicron. Would be much more compelling if this study including much more recent data and looked at whether the lag was consistent despite changing variants and changing treatments. And, the availability of outpatient treatments like Paxlovid may be game changing."

Authors' reply to the Reviewer:

When we have extended the study period, we see that the about 18 days lag is consistent in the ancestral variants, but during Omicron our analysis reveals a shorter time period, which could be explained by the fact that Primary Care facilities were overloaded at that time. We leave the comparison of earlier variant waves to Omicron for future research.

Unfortunately, Paxlovid was not introduced until very recently (a few days ago) in Spain, following the European Medicines Agency approval, so its effect cannot be assessed.

Changes included in the manuscript:

Table 2 presents a summary of anticipation time in days depending on the data window analysis.

The following sentence has been included in the results. "*Table 2 extends the data timeframe. We present estimates of anticipation lag time in days and the corresponding CI, using a monthly timeframe rolling window until March 2022*".

The following sentence has been included in the discussion section. *"With Omicron our analysis reveals a shorter anticipation lag in days, which could be explained by the fact that Primary Care facilities were overloaded at that time. We have considered the availability of outpatient treatments like Paxlovid may be game changing, but we have ruled out its effect because Paxlovid was approved by the European Medicines Agency only on January 28, 2022 and introduced in Spain very recently."*
